# Exploring the core network of the structural covariance network in childhood absence epilepsy

**DOI:** 10.1016/j.heliyon.2023.e22657

**Published:** 2023-11-22

**Authors:** Merel J.A. Eussen, Jacobus F.A. Jansen, Twan P.C. Voncken, Mariette H.J.A. Debeij-Van Hall, Jos G.M. Hendriksen, R. Jeroen Vermeulen, Sylvia Klinkenberg, Walter H. Backes, Gerhard S. Drenthen

**Affiliations:** a Department of Biomedical Technology, Eindhoven University of Technology, Eindhoven, the Netherlands; b Department of Electrical Engineering, Eindhoven University of Technology, Eindhoven, the Netherlands; c Department of Radiology & Nuclear Medicine, Maastricht University Medical Center, Maastricht, the Netherlands; d School for Mental Health and Neuroscience, Maastricht University Medical Center, Maastricht, the Netherlands; e Department of Neurology, Maastricht University Medical Center, Maastricht, the Netherlands; f Epilepsy Center Kempenhaeghe, Heeze, the Netherlands

**Keywords:** Childhood absence epilepsy, Cognitive performance, cortical thickness, graph theory, structural covariance network, assortativity

## Abstract

Childhood absence epilepsy (CAE) is a generalized pediatric epilepsy, which is generally considered to be a benign condition since most children become seizure-free before reaching adulthood. However, cognitive deficits and changes of brain morphological have been previously reported in CAE. These morphological changes, even if they might be very subtle, are not independent due to the underlying network structure and can be captured by the structural covariance network (SCN).

In this study, SCNs were used to quantify the structural brain network for children with CAE as well as controls. Seventeen children with CAE (6-12y) and fifteen controls (6-12y) were included. To estimate the SCN, T1-weighted images were acquired and parcellated into 68 cortical regions. Graph measures characterizing the core network architecture, i.e. the assortativity and rich-club coefficient, were calculated for all individuals. Multivariable linear regression models, including age and sex as covariates, were used to assess differences between children with CAE and controls. Additionally, potential relations between the core network and cognitive performance was investigated.

A lower assortativity (i.e. less efficiently organized core network organization) was found for children with CAE compared to controls. Moreover, better cognitive performance was found to relate to stronger assortative mixing pattern (i.e. more efficient core network structure). Rich-club coefficients did not differ between groups, nor relate to cognitions.

The core network organization of the SCN in children with CAE tend to be less efficient organized compared to controls, and relates to cognitive performance, and therefore this study provides novel insights into the SCN organization in relation to CAE and cognition.

## 1 Introduction

Childhood absence epilepsy (CAE) is a common form of idiopathic generalized epilepsy (IGE) in school-aged children [1]. Since most children become seizure-free before reaching adulthood, CAE is thought to be a benign condition. Moreover, since large structural abnormalities are generally not regarded to be part of the pathophysiology of CAE, MRI is often omitted from the standard clinical workup. However, CAE is accompanied by various cognitive impairments [[2], [3], [4]], as well as cerebral alterations ranging from morphological changes [[5], [6], [7]] to functional and structural network changes [[8], [9], [10], [11]]. These findings suggest that both morphological and network alterations might be part of the underlying pathology of CAE. Moreover, the morphological changes are not independent due to the underlying network structure. The structural covariance network (SCN) concept has the potential to capture the underlying network structure using morphological correlations, including subtle morphological alterations, and therefore might have a lot of potential in CAE research.

The theory behind SCN rests on the assumption that connected regions, either functionally connected or connected via white-matter tracts, share similar patterns of morphology since they have common developmental, maturational, and pathological influences [12]. Accordingly, the distance-based SCN captures the interregional correlations of a morphological property, such as the cortical thickness, across a group of subjects. Since the distance-based SCN is calculated on morphological properties across a group of subjects it hampers direct estimation of individual network characteristics. To overcome this limitation, Saggar et al. [13] introduced an individual SCN method, the add-one-patient approach, which has already shown value in epilepsy research [13,14]. Moreover, a prior study expanded upon this approach by introducing an additional reference group to obtain individual SCNs for both patients and controls (i.e. add-one-participant) [15]. Thus, facilitating the analysis of individual network characteristics of all participants and their relation to cognitive performance. There are multiple approaches to construct individual SCNs, and each method can yield distinct and valuable information. For example, Tijms et al. [16] introduced the cube-based method, which directly estimates within-subject connectivity, while the distance-based method is based on individual variations with respect to a group of subjects, i.e. the reference group. Therefore, changes in morphology with respect to the healthy reference group relates to a loss of connection strength in the distance-based SCNs, making it especially sensitive to network alterations due to disease-related cortical changes.

Since CAE is a generalized epilepsy, we aim to describe the organization of the SCNs in terms of the global brain network. Prior research has identified that a core network of common regions is involved in the occurrence of absence seizures [17,18]. Therefore, in this paper we focus on graph measures that quantify the core structure of a network, i.e. assortativity and rich-club coefficient. Assortativity is a measure that relates to the connectivity pattern of all nodes in the network and serves as an indirect measure of network resilience. In an assortative network, nodes with a similar degree are more likely to be interconnected. In this case, high-degree nodes tend to connect to other high-degree nodes, making the network more robust to local damage [19,20]. Furthermore, an assortative network implies efficient information flow through the brain. Moreover, networks with efficient global communication entail a rich-club organization, in which a subnetwork of relatively high-degree nodes, so-called hubs, play a crucial role. These hubs tend to be highly interconnected forming a highly interconnected club, the so-called rich-club [7].

We hypothesize that children with CAE show alterations in their SCNs compared to healthy controls, expressed by a lower assortativity and rich-club organization, since these indicate a less efficient global network organization. Moreover, changes in the core network of the SCN of children with CAE are expected to relate to worse cognitive performance compared to healthy controls. The current study aims to explore the potential relation between disrupted core network organization of the SCN and cognitive performance in children with CAE.

## 2 Materials and methods

### 2.1 Participants

Seventeen children with CAE (6-12y, 12 male) and 15 healthy control subjects (6-12y, 11 male) were included [6,8]. The children with CAE were clinically diagnosed in accordance with ILAE statements for CAE [21,22]. Only children with early CAE (within 2 years of diagnosis) were recruited via pediatric neurologists of the Kempenhaeghe Epilepsy Centre and the Maastricht University Medical Centre. Exclusion criteria for participation in our study were; 1) a diagnosis other than CAE according to the ILAE criteria, 2) Intellectual disability or other diseases/causes that may underlie cognitive impairment, 3) History of major head trauma or head/brain surgery and 4) MRI lesions on (previous) structural brain MRI- or CT-scans or symptomatic epilepsies (e.g. epilepsy related to tumours, vascular abnormalities, congenital dysgenesia). More details on inclusion and exclusion criteria can be found in a previous publication [6]. All caregivers and participants over 12 years of age gave written permission prior to inclusion. This research was approved by the local medical ethics committee azM/UM NL55455.068.15/METC152055 and the research protocol is listed at clinicaltrials.gov under NCT02954107. Demographic data were collected and all subjects completed neuropsychological assessment. For each subject, general intelligence was estimated from four WISC-III subtests (similarities, vocabulary, picture completion and block design) and processing speed index was evaluated based on two subsets of WISC-III (coding, and symbol search) [23] by an experienced neuropsychologist from Kempenhaeghe (JGMH). Both cognitive measures were expressed as standard scores relative to normative data, with a mean processing speed index of 100 (SD = 15) and general intelligence having a mean of 10 (SD = 3). The processing speed index of the controls was significantly higher compared to the CAE group (107.7 ± 13.9 vs. 95.4 ± 15.4, p < .03). Additionally, for the SCN analysis, 22 healthy controls were included as a reference group (8-12y, 9 male) [24]. The subject characteristics are shown in Table 1.

**Table 1:** Subject characteristics of children with CAE, healthy controls, and subjects included in the reference group.

	CAE	Controls	p-value	Reference group
Sample size (N)	17	15	–	22
Age (y, median (IQR))	8.6 (4.1)	9.8 (3.3)	.36	10.3 (1.5)
Sex (M/F)	12/5	11/4	.86	8/13
WISC-III				
General intelligence (mean ± SD)	9.6 ± 1.9	10.9 ± 2.3	.09	–
Processing speed index (mean ± SD)	95.4 ± 15.4	107.7 ± 13.9	**.03**	–
Age of onset (years, mean ± SD)	8.0 ± 2.0	–	**-**	–
Duration of epilepsy (years, mean ± SD)	1.2 ± 0.7	–	**-**	–
Total duration AED use (weeks)	27 ± 26	–	**-**	–
Schooling (regular/special)	16/1	15/0	.34	–
Family history of epilepsy (Yes/No)	2/15	1/14	.62	–

Y, years; SD, standard deviation; CAE, childhood absence epilepsy; WISC-III, Wechsler Intelligence Scale for Children, third edition; AED, anti-epileptic drugs.

### 2.2 MRI acquisition

All participants were scanned on a 3.0T MR scanner (Philips Achieva, Best, the Netherlands) using a 32-element phases-array coil. Structural MR images were acquired for each subject using a T1-weighted three-dimensional turbo field echo sequence (repetition time (TR) = 8.4 ms, echo time (TE) = 3.8 ms, flip angle (FA) = 8°, voxel size 1x1x1 mm). Furthermore, for the reference group, structural T1-weighted images were also acquired with a turbo field echo sequence (TR = 8.1 ms, TE = 3.7 ms, FA = 8°, voxel size 1x1x1 mm).

### 2.3 Structural covariance network construction

As part of the FreeSurfer pipeline (version 7.1.0) [25], the cortical surface was automatically reconstructed and parcellated into 68 regions based on the Desikan-Killiany atlas [26]. Subsequently, the inner and outer cortical surfaces were visually inspected and manually corrected, followed by a re-computation in FreeSurfer. The cortical thickness of each parcellated region was determined by calculating the mean cortical thickness over the area. Cortical thickness decreases with age, differs across the sexes, and scales with intracranial volume [[27], [28], [29]]. Therefore, potential effects of age, sex, and total intracranial volume on the cortical thickness were accounted for by correcting the cortical thickness values of each region using a linear regression model [30].

The correlations of the corrected cortical thicknesses over a group of participants between all possible pairwise regions were calculated, using Pearson's correlation coefficient. The SCN captures the connectivity between every regional pair represented in an adjacency matrix. To obtain individual SCNs for both the children with CAE and the healthy controls using the add-one-participant approach, an additional reference group is needed [13,15]. Hence, each individual (i.e. CAE or control group) is added to the reference group prior to calculating the SCN. Only statistically significant positive correlations were included in the SCN. The role of negative correlations is still unclear and therefore not included in the SCN [31]. The individual SCN analysis was performed using in-house MATLAB code, made publicly accessible on GitHub (https://github.com/GSDrenthen/SCN).

The graph measures characterizing the brain network are influenced by the total number of connections in the SCN [32]. Therefore, to ensure our results are not biased by the number of connections, we only compare SCNs with equal numbers of connections, i.e. networks that are equally sparse. In this research, the SCNs are evaluated across a range of sparsity values (72.5 %–90 %) with an interval of 0.5 %. The sparsity ranges from a network solely including those significantly positive connections (72.5 %) to the upper boundary (90 %), which is chosen to prevent networks to contain too many disconnected nodes which could hamper further graph analysis.

### 2.4 Network analysis

**Fig. 1:**
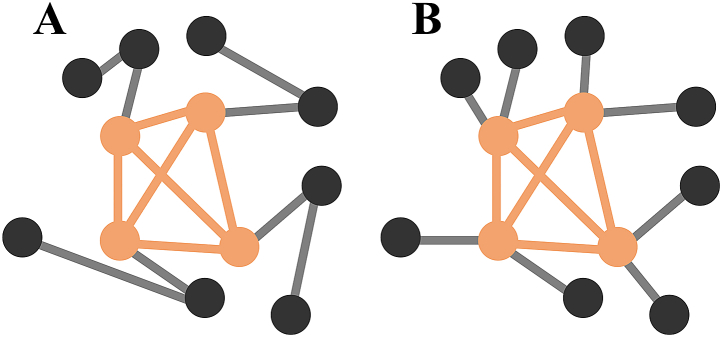
Graphical representation showing the distinction between rich-club and assortativity. A) and B) both contain a rich-club (orange group), however A) is an assortative network (r = 0.5), whereas B) is a disassortative network (r = −0.4).

Using the Brain Connectivity Toolbox [20] and in-house developed code in MATLAB (R2020a, MathWorks, Natick, MA, USA) the assortativity and rich-club coefficients are calculated for each network. Schematic illustrations of an assortative network, and a disassortative network are shown in Fig. 1A and B respectively.

Assortativity provides insight into the core structure of the network, and is an indirect measure of resilience, as it indicates the network's vulnerability to the removal of high-degree nodes. Assortativity is defined as the correlation of a nodal property, typically the nodal degree, between the nodes on two opposite ends of a connection (Equation (1)) [19].

(1) $$r=\frac{\frac{1}{M}\sum_{i}j_{i}k_{i}-{\left[\frac{1}{M}\sum_{i}\frac{1}{2}\left(j_{i}+k_{i}\right)\right]}^{2}}{\frac{1}{M}\sum_{i}\frac{1}{2}\left({j_{i}}^{2}+{k_{i}}^{2}\right)-{\left[\frac{1}{M}\sum_{i}\frac{1}{2}\left(j_{i}+k_{i}\right)\right]}^{2}}$$

with *j*_*i*_ and *k*_*i*_ being the nodal degrees at the ends of connection *i*, with *i=1, …, M*. The assortativity ranges from −1 to 1, where a negative or positive value indicates a disassortative or assortative network, respectively. In an assortative network the nodes are likely to connect to similar degree nodes. In such a network, high-degree nodes contribute to a strong and resilient core structure.

The rich-club coefficient (RCC) is another measure of the core network structure. In contrast to the assortativity index, where the nodal degree of all regions is considered, the RCC is calculated by considering a subnetwork consisting of hubs only. A node is considered a hub when the nodal degree exceeds threshold *k,* which is the mean plus one standard deviation of the degree in the reference group's SCN*.* A network has a high RCC when the high-degree nodes (i.e. hubs) are also highly interconnected amongst each other. The existence of a rich-club is important for efficient information processing across segregated regions.

The rich-club coefficient is calculated as Equation (2) [33].

(2) $$RCC\left(k\right)=\frac{2{*E}_{>k}}{N_{>k}\left(N_{>k}-1\right)}$$

with *N* > _*k*_ being the number of hubs, and *E* > _*k*_ denotes the number of existing connections in the subnetwork. Hence, the denominator represents the number of total possible connections between the hubs in the subnetwork. Subsequently, the coefficient requires normalization with respect to random networks, since high-degree nodes have a higher probability of being interconnected by chance. Hence, normalization is performed by calculating the rich-club coefficients for 1000 random networks (RCCnorm(k)=RCC(k)/RCCrand(k)). Rich-club organization is present in the network when the normalized coefficient is larger than 1, and a higher RCC indicates stronger interconnected hubs.

### 2.5 Statistical analysis

Potential group-differences in general intelligence and processing speed index were assessed by the independent-samples *t*-test. As age was not normally distributed, age differences were assessed using the non-parametric Wilcoxon-rank sum test. Differences in sex, schooling and history of epilepsy were tested with the Chi-squared test of independence.

Cortical thickness was not normally distributed in every region, therefore to test for differences in cortical thickness between the children with CAE and the healthy control group, Wilcoxon-rank sum tests were used for each of the 68 regions. The results were corrected for multi comparisons using the false discovery rate (FDR) [34].

To test for between-group differences in both graph measures between the children with CAE and controls, multivariable linear regression models with sex and age as covariates were used. Since the residuals of the linear model were not normally distributed, Box-Cox transformations were applied for both the graph measures at each sparsity value [35]. After Box-Cox transformation, the residuals were normally distributed. A model for each graph measure was fitted at each sparsity.

Furthermore, linear regression models were also used to assess the potential relationship between individual graph measures and cognitive performance, while correcting for age, sex, and diagnostic group (i.e. CAE vs controls). Respectively, general intelligence and processing speed index were investigated.

The potential influence of the duration of anti-epileptic drug (AED) use on both the average cortical thickness as well as on the assortativity was evaluated through linear multivariable regression models for the CAE group, with age and sex added as covariates.

All statistical analysis were performed using MATLAB (R2020a, MathWorks, Natick, MA, USA), and statistical significance was inferred at p < .05.

## 3 Results

Regional cortical thicknesses did not differ significantly between the children with CAE and controls (after FDR correction).

**Fig. 2:**
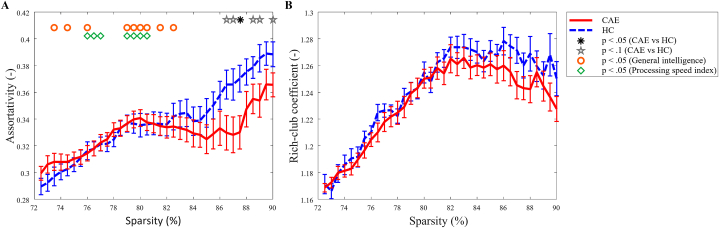
A) Assortativity, B) Rich-club coefficient. Mean and standard errors for children with CAE (solid) and healthy controls (dashed) are plotted as a function of sparsity. The asterisk indicates a significant between-group difference (p < .05) at the corresponding sparsity and the star a trend (p < .10). The significant relationships between graph measure and subject characteristics are denoted by symbols: ○general intelligence and ◊ processing speed index.

The mean and standard error of the assortativity and RCC are shown as a function of sparsity in Fig. 2A and B respectively. The asterisk indicates a significant between-group difference (p < .05) and the star a trend towards significance (p < .10), whereas the additional symbols denote significant relationships between the graph measure and either general intelligence or processing speed at the corresponding sparsity (p < .05).

**Fig. 3:**
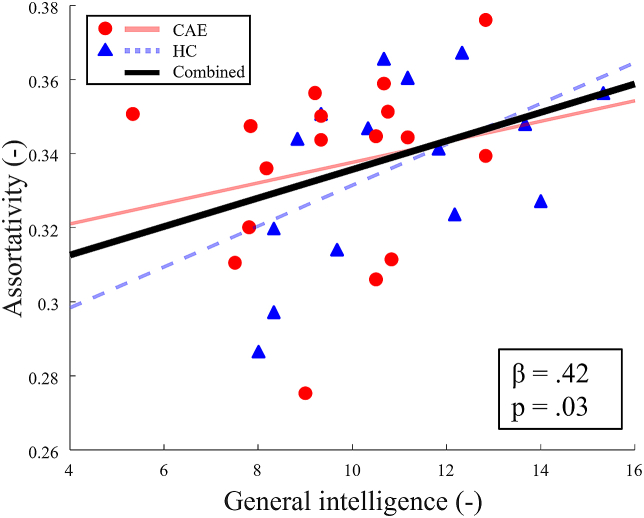
A representation of the relation of assortativity vs. general intelligence at a network sparsity of 79 %. Linear least-squares lines are fitted through the data points for visualization, with the black line representing the tested relation of both groups combined. Standardized regression coefficients (β) and corresponding p-values are shown.

The mean assortativity as a function of the sparsity was positive for both groups, indicating that all networks were organized in an assortative mixing pattern as opposed to a disassortative pattern. A significant between-group difference was found for a single sparsity value of 87.5 % (β = −0.41, p = .03), indicating a less assortative network in the children with CAE compared to the controls. Moreover, trends towards significance (p < .10) were found for sparsity values 86.5 %, 87 %, 88 %, 88.5 % and 90 %. Furthermore, for the combined CAE and control group, both general intelligence and processing speed related significantly to assortativity. Fig. 2A shows the sparsity values for which positive associations were found. For the networks with a representative sparsity of 79 %, a positive association with processing speed index (β = 0.44, p = .04) was found. For illustrative purposes, the assortativity of the 79 % sparse networks as a function of general intelligence is visualized in the scatter plot in Fig. 3.

On average, for both groups the networks exhibited a rich-club organization (RCC>1). Although the rich-club coefficient was lower in children with CAE for most sparsity values, it does not reach significance. Moreover, no significant associations were found between cognitive performance and RCC.

Using regression, no significant relations were found between average cortical thickness and assortativity values and the duration of AED use in the CAE group (p > .10).

## 4 Discussion

### 4.1 Current findings

In this study, we investigated whether the core network organization of the SCN was altered in children with CAE compared to a control group. Moreover, we studied whether these organizational alterations were related to cognitive performance in terms of general intelligence and processing speed. We found less efficient core network organization in children with CAE compared to the control group. Furthermore, a stronger assortative mixing pattern was related to higher cognitive performance.

### 4.2 Differences in regional cortical thickness

Widespread cortical thickness differences were not expected, since large structural abnormalities are not part of the pathophysiology of CAE. Nevertheless, a previous study reported increased cortical thickness of the right posterior cingulated gyrus and the left medial occipital in children with CAE compared to controls [36]. In this study, we did not find significant regional differences between both groups. Therefore, the results of the SCN analyses in our study are not driven by large differences in anatomical morphology. More importantly, it shows that although subtle differences between the groups are not detectable on a regional level, the SCNs have the potential to express these changes on a network level.

### 4.3 Structural covariance network construction

Thus far, only one study has previously investigated group-level SCNs concerning CAE [37]. Their population included young adults whose last seizure occurred on average 9 years ago. Furthermore, 73 % of the subjects had a complete remission of seizures, while not receiving antiepileptic drugs (AEDs) for at least two years. Their study demonstrated long-term differences in connectivity patterns between CAE subjects and controls using group-level networks. However, the group-level approach hampers analysis of individual network characteristics. The current study is the first that obtained SCNs on an individual level for both children with CAE and controls. Hence, this is the first time that subject characteristics of these individuals can be associated with individual-based graph measures.

### 4.4 Assortativity

The tendency of nodes to connect with similar degree nodes is expressed with the assortativity index. In an assortative network, fast information spread through the network is facilitated by the core of the network. The current study found assortative mixing patterns in the networks of children with CAE as well as in the networks of the controls. The assortativity in networks of children with CAE was on average lower than controls, which suggests that similar degree nodes in children with CAE tend to be less often interconnected. However, only for sparse networks (i.e. the core network) this effect revealed differences trending towards significance, and therefore, this should be interpreted with caution. These findings might suggest slower information spread in the CAE group, which might be a mechanism for the containment of epileptic seizure activity [19]. Furthermore, we revealed that a decrease in assortative mixing pattern was related to worse cognitive performance. An impaired assortative mixing pattern might result in a less efficient information spread and thus a slower information spread. This suggests that a less efficiently organized network impacts a lower cognitive performance. Moreover, since cognitive problems are considered a part of the CAE syndrome, these results combined indicate that assortativity might reflect a possible underlying mechanism of CAE-related cognitive impairments.

Another property of assortative mixing networks is that they are more resilient to failure of high-degree nodes compared to disassortative mixing networks, since in such an event the information flow remains intact via other high-degree nodes. Relative to controls, the high-degree nodes of the core networks of children with CAE might be a bit more distributed and therefore the network could be more vulnerable to node failures which potentially results in disrupted network connectivity. Moreover, given the relation between assortativity and cognitive performance, nodal failure might be part of the reason why cognitive performance is lower when the assortative mixing pattern is impaired.

### 4.5 Rich-club

While assortativity is a graph measure describing the global organization of all nodes, the rich-club coefficient focuses on a subnetwork including only the hubs. We found that the networks of children with CAE and controls both contain a rich-club organization. The benefit of this organization is that, regardless of nodal failure, efficient information processing is not compromised. However, while the rich-club coefficient appears to be slightly lower in children with CAE compared to the control group, this did not reach significance. Similarly, the rich-club organization did not relate to cognitive performance. Therefore, the assortativity index, which also includes the lower degree nodes, seems to be a core-network measure that is more sensitive to CAE.

### 4.6 Study considerations

The main limitation of this study is the relatively low sample size. However, despite our limited sample, we have already identified both between-group differences as well as associations with subject characteristics. While the results of the current study already show the potential of SCNs, it is still too preliminary to make individual clinical predictions of disease outcome. Further studies with larger sample sizes are recommended to further investigate the individually estimated SCNs, and to obtain more statistical power. Additionally, longitudinal data and seizure characteristics, such as seizure frequency and the time until reaching seizure control, may gain more insights into the persistence of structural network alterations despite seizure control.

The broad availability of T1-weighted images contributes to a wide application of SCNs, moreover, this modality is beneficial in terms of cost-efficiency and acquisition time, which is especially relevant when scanning people that are unable to endure long scanning times, such as children, elderly, and people with movement disorders or claustrophobia. Furthermore, the T1-weighted derived SCN networks are a promising tool compared to diffusion-tensor imaging (DTI). A prior study by Gong et al. showed that 35–40 % of all pairwise connected cortical regions in the SCN have a matching diffusion-derived connection in DTI [31]. This suggests that both structural modalities, contain complementary information, and thus that the SCN contains additional biological mechanisms, aside from the direct fiber connections.

Previous studies showed morphological alterations, i.e. cortical thinning related to anti-seizure medication such as valproate, and cognitive performance changes were associated with the various AEDs [4,[38], [39], [40]]. Hence, we cannot rule out the possibility that AEDs can impact our results, although large influences of AEDs on cortical thickness are not expected due to a relative short duration of AED use. Furthermore, the AEDs are prescribed according to the current guidelines for CAE. Therefore, our results are based on a representative sample for children with CAE at this point in time. Moreover, no relation between AED use and either cortical thickness or assortativity was found. Therefore, we expect that the potential effect of medication use on our results is minimal.

Even though the T1-weighted images of the reference group were acquired with slightly different scan parameters, our results are not biased by these differences. While the cortical thickness of the reference group serves as a constant factor to construct the SCNs, no direct comparisons between the reference group and the study cohort (i.e. CAE and controls) were made. Furthermore, the reference group was slightly older, however, age was accounted for in the preprocessing where the cortical thickness was corrected for the age of the reference group as well.

## 5 Conclusion

This exploratory research shows that the structural networks in children with CAE tend to be less efficiently organized compared to healthy controls and that they are related to cognitive performance. Therefore, CAE may be considered a brain network disorder, with less efficient networks potentially playing a role in the underlying pathology of CAE.

## Declarations

### 5.1 Ethics statement

This study was reviewed and approved by local medical ethics committee azM/UM, with the approval number: NL55455.068.15. All participants (or their proxies/legal guardians) provided informed consent to participate in the study.

## Data availability statement

Data will be made available on request.

## CRediT authorship contribution statement

**Merel J.A. Eussen:** Formal analysis, Visualization, Methodology, Writing - original draft. **Jacobus F.A. Jansen:** Conceptualization, Methodology, Supervision, Writing - review & editing. **Twan P.C. Voncken:** Data curation, Writing - review & editing. **Mariette H.J.A. Debeij-Van Hall:** Data curation, Conceptualization, Writing - review & editing. **Jos G.M. Hendriksen:** Data curation, Writing - original draft. **R. Jeroen Vermeulen:** Data curation, Writing - review & editing. **Sylvia Klinkenberg:** Conceptualization, Data curation, Writing - review & editing. **Walter H. Backes:** Conceptualization, Writing - review & editing. **Gerhard S. Drenthen:** Conceptualization, Methodology, Software, Supervision, Formal analysis, Writing - review & editing.

## Declaration of competing interest

The authors declare that they have no known competing financial interests or personal relationships that could have appeared to influence the work reported in this paper.
